# The polygenic and reactive nature of observed parenting

**DOI:** 10.1111/gbb.12874

**Published:** 2023-11-29

**Authors:** Jana Runze, Marian J. Bakermans‐Kranenburg, Charlotte A. M. Cecil, Marinus H. van IJzendoorn, Irene Pappa

**Affiliations:** ^1^ Research Institute of Child Development and Education University of Amsterdam Amsterdam the Netherlands; ^2^ ISPA – University Institute of Psychological, Social and Life Sciences Lisbon Portugal; ^3^ Center for Attachment Research The New School New York New York USA; ^4^ Department of Psychology Stockholm University Stockholm Sweden; ^5^ Department of Child and Adolescent Psychiatry Erasmus Medical Center Rotterdam the Netherlands; ^6^ Department of Epidemiology Erasmus Medical Center Rotterdam the Netherlands; ^7^ Department of Psychology, Education and Child Studies Erasmus University Rotterdam Rotterdam the Netherlands; ^8^ Research Department of Clinical, Education and Health Psychology Faculty of Brain Sciences, UCL London UK; ^9^ Clinical Child and Family Studies VU University Amsterdam Amsterdam the Netherlands

**Keywords:** educational attainment, evocative gene–environment correlation, maternal sensitivity, parenting behavior, polygenic risk score (PGS)

## Abstract

In Wertz et al. (2019), parents' polygenic scores of educational attainment (PGS‐EA) predicted parental sensitive responses to the child's needs for support, as observed in a dyadic task (i.e., observed sensitivity). We aimed to replicate and expand these findings by combining longitudinal data, child genotype data and several polygenic scores in the Generation R Study. Mother–child dyads participated in two developmental periods, toddlerhood (14 months old; *n* = 648) and early childhood (3–4 years old, *n* = 613). Higher maternal PGS‐EA scores predicted higher observed sensitivity in toddlerhood (*b* = 0.12, 95% CI 0.03, 0.20) and early childhood (*b* = 0.16, 95% CI 0.08, 0.24). Child PGS‐EA was significantly associated with maternal sensitivity in early childhood (*b* = 0.11, 95% CI 0.02, 0.21), and the effect of maternal PGS‐EA was no longer significant when correcting for child PGS‐EA. A latent factor of PGSs based on educational attainment, intelligence (IQ) and income showed similar results. These polygenic scores might be associated with maternal cognitive and behavioral skills that help shape parenting. Maternal PGSs predicted observed sensitivity over and above the maternal phenotypes, showing an additional role for PGSs in parenting research. In conclusion, we replicated the central finding of Wertz et al. (2019) that parental PGS‐EA partially explains parental sensitivity. Our findings may be consistent with evocative gene–environment correlation (rGE), emphasizing the dynamic nature of parenting behavior across time, although further research using family trios is needed to adequately test this hypothesis.

## Graphical abstract

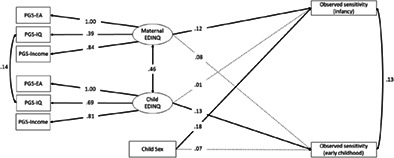

## 1 INTRODUCTION

Parenting is a complex phenotype, ranging from sensitive responsiveness and limit‐setting to harsh and neglecting approaches, and it is supposed to substantially shape child development. Previous studies on (mostly mothers') sensitive responses toward the child (i.e., maternal sensitivity) documented the predictive, positive associations with children's cognitive and language development, self‐regulatory executive functioning, socio‐emotional development and less externalizing behavior problems.^1^, ^2^, ^3^, ^4^, ^5^ Antecedents of parenting such as parental socioeconomic status, mental health and experiences of adversities have been found to explain part of the variance in sensitivity^6^ but the direction of effects is not always clear. In this study, we use a polygenic score approach to investigate the role of educational attainment, intelligence and income in predicting maternal sensitivity. These polygenic scores are assumed to refer to cognitive problem solving abilities as well as to noncognitive abilities implied by educational and social success, such as planning skills, task persistence or stress regulatory abilities.^7^ Cognitive and noncognitive abilities are represented by the PGSs for IQ, educational achievement and income. The PGS of IQ is thought to tap more into the cognitive abilities and the PGSs of educational attainment and income are thought to represent a mixture of cognitive and noncognitive abilities. However, a sharp division of these cognitive and noncognitive abilities in PGSs is complicated, although the GWAS‐by‐subtraction approach is promising.^8^ Cognitive and noncognitive abilities may both be relevant for effective parenting.

Previous (mostly child‐based) twin studies on various dimensions of observed or self‐reported parenting have shown moderate genetic effects, especially for reported parenting.^9^, ^10^ More similar parenting behavior in families with monozygotic versus dizygotic same‐sex twins might be interpreted as evocative child effects on parental sensitive limit‐setting,^9^ and a meta‐analysis documented substantial evocative gene–environment correlations for parental positive reactions (e.g., structuring and sensitivity^11^). However, molecular genetics studies examining candidate genes to explain variance in parenting showed equivocal results, and the main effects of (sets of) candidate genes seemed difficult to replicate.^12^ The introduction of polygenic scores (PGSs) based on genome‐wide association studies (GWAS) incorporating numerous single nucleotide polymorphisms (SNPs) is a promising development in the area of parenting research because multiple genes are thought to be involved in the complex phenotype of parenting. Indeed, one study showed evidence for SNP heritability (*h* ^2^_SNP_ = 0.10, 95% CI 0.00, 0.19, *N* = 6453) of self‐reported parenting using genome‐wide complex trait analysis (GCTA),^13^ a tool that estimates the variance explained by all SNPs instead of any particular SNP.^14^ Conducting a GTCA however requires large samples and even the ALSPAC cohort in which the study was performed was deemed to be underpowered for this approach.^13^

An alternative methodology is the application of PGSs derived from the published combined results of consortia with GWAS data on hundreds of thousands of participants. In the ground‐breaking Dunedin study with observed parenting and parental GWAS data,^15^ the authors used the PGS for educational attainment (PGS‐EA) based on GWAS data of more than a million participants^16^ to predict variance in parenting in their sample of 702 participants. They found that parents with higher PGS‐EA provided more warm, sensitive and stimulating parenting to their 3‐year‐old children. Part of this association, however, might be evocative child effects as children inherit parental genes that might lead to children's phenotypical traits (e.g., aggression) that trigger specific parenting behavior (e.g., harsh limit setting). In the Dunedin study, the children's genomes were not assessed, and the authors tested for child effects indirectly, deriving temperament‐like child traits from video‐recorded child interactions with the parent. Based on this temperament measure, they found no evidence of evocative child effects. In a follow‐up study, the authors used data from the E‐Risk study, where observed parenting and genetic data of both parent and child were available. In that study, evocative gene–environment correlations between children's genetics and dimensions of parenting were found.^15^ The divergent findings may result from different measures (observed child behavior versus genetic child data) or other factors (e.g., age of the child at the time of assessment). Additional studies are required to clarify the role of genetics in parenting and the extent of child genetic effects on parenting.

In the current study, we aimed to replicate the main finding of the Dunedin study, in particular the effect of the maternal PGS‐EA on observed parenting, measured at two developmental time points, namely in toddlerhood (14 months) and early childhood (3 and 4 years). Maternal sensitivity at its core generally shows continuity during development,^17^ but there is evidence that maternal sensitivity is also adaptive to changes in child development and may vary across time,^18^ likely in response to the child's needs and behaviors. To better capture the dynamic nature of maternal sensitivity, we included two developmental periods that are characterized by rapid changes in child's needs and challenge parenting behavior in a different way.

In addition, we controlled for evocative child PGS‐EA effects extracted from the children's genomes. Next, we extended our search for polygenic effects on observed parenting by adding other relevant maternal PGSs, namely the PGSs of general intelligence (PGS‐IQ) and income (PGS‐income). Low maternal education, general intelligence and income have previously been associated with lower maternal sensitivity,^19^, ^20^ while higher maternal education and income are correlated with more supportive and sensitive parenting.^21^ Supportive and sensitive parenting refer to interactions in which parents are aware of their child's emotional and physical needs and respond appropriately and consistently. Using the additional genetic indicators for intelligence and income to predict observed parenting we expected to better capture the complexity of parenting. These three PGSs of EA, IQ and income are expected to depict effects of both cognitive and what have been collectively called “noncognitive skills.”^8^ The “noncognitive skills” refer to factors such as planning skills, task persistence or delay of gratification and stress regulatory abilities, that are considered to be equally important as IQ in explaining academic and employment outcomes.^22^ This study focuses on the effects of the cognitive and related but not purely noncognitive domains on parenting. This focus on highly related components of socio‐intellectual functioning is of course a restriction considering the large number of PGSs for affective dimensions of functioning. In our opinion, this selection has the advantage of limiting the number of statistical tests and is underrepresented in parenting literature, where the focus often lies on the socio‐emotional domain (i.e., mood and emotions of the parents). Finally, we used the relevant maternal phenotypes (i.e., educational level, IQ and income) to test for associations with observed parenting and investigated whether the PGSs had any additional predictive power over and above the maternal phenotypes.

In sum, we aimed to replicate and extend the Dunedin findings, using data from the Generation R Study, a population‐based prospective cohort study based in The Netherlands in which observational parenting data were available at two developmental time points, toddlerhood (14 months) and early childhood (3–4 years).^23^ Our first aim was to test the associations of maternal PGS‐EA with observed sensitive parenting. We also tested whether child genetics explained part of these associations. Our second aim was more exploratory, in that we investigated whether including parental PGSs of IQ and income were associated with observed parenting and had predictive power over and above maternal education level, IQ and income.

## 2 MATERIALS AND METHODS

### 2.1 Setting

The mothers and children in this study were participants of the Generation R Study, a population‐based prospective cohort study based in Rotterdam, the Netherlands.^23^ Mothers with a delivery date between April 2002 and January 2006 were enrolled in the Generation R Study. The Medical Ethical Committee of the Erasmus Medical Centre approved the study protocol; data collection and ethical issues were described in detail elsewhere. The study was preregistered at https://doi.org/10.17605/OSF.IO/2EN8Y.

### 2.2 Study population

A subgroup of 1247 women and their children were invited to our research center for observational assessments during infancy and toddlerhood. This group (Generation R Focus cohort) is of Dutch ethnic origin.^24^ The mean age of the mothers in our sample was 31 years (SD = 4.49). Twenty‐five mothers had twins: in these cases, one sibling of each twin pair was randomly selected for analyses. No siblings or other relatives participated in this study. 56% (*n* = 704) of mothers and children participated in laboratory observations to assess maternal sensitivity at age 14 months. 59% (*n* = 740) participated in laboratory observations at 3 years and home visits at 4 years.

Non‐response analyses showed that dyads included in the analyses did not differ from the excluded dyads on child sex, maternal educational level and maternal sensitivity.^25^

### 2.3 Observed maternal sensitivity

Maternal sensitivity was observed first during a lab visit at the child's age of 14 months (during free play),^26^ and then during a lab visit at the age of 3 years and a home visit at age 4 years (with two tasks: building a tower and etch‐a‐sketch).^25^ Maternal sensitivity was coded with satisfactory intercoder agreement from video recordings (free play ICC = 0.79; tower task ICC = 0.75; etch‐a‐sketch ICC = 0.79).^26^ An overall score for maternal sensitivity in early childhood was computed by combining the 3‐ and 4‐year measurements, as previously described.^25^

### 2.4 Maternal education, income and IQ

Information about maternal education was obtained by questionnaire during enrollment in the Generation R Study and categorized as follows: high (34.9%, university degree), mid‐high (24.5%, higher vocational training), mid‐low (25.9%, >3 years general secondary school, intermediate vocational training) and low (14.7%, primary school, lower vocational training, intermediate general school or 3 years of less general secondary school). Information about net household income (76.0% > €2200 per month) was obtained by postnatal questionnaires completed by both parents.

Maternal IQ was measured when the children were around their sixth birthday (mean age = 6.0 ± 0.3 years) at the Generation R research center. Maternal non‐verbal IQ was assessed using a computerized version of the Ravens Advanced Progressive Matrices Test (APM), set I.15.^27^ The mean intelligence score was 100 (SD = 15) for the whole Generation R sample, as expected.

### 2.5 Genotyping and imputation

A detailed description of the Generation R Biobank has been published.^28^ Maternal blood samples were available for 1247 mothers of the Generation R Focus cohort. All mothers were of European ancestry, confirmed using principal components analysis on GWAS data. DNA was genotyped using the Infinium Global Screening Array with Multi‐Disease drop‐in (GSA‐MD), version 2. Child blood samples were collected from cord blood at birth (Illumina 610 K Quad Chip) or from venipuncture during a lab visit at around 6 years (Illumina 660 K Quad Chip). The Illumina 610 K and 660 K were merged based on their overlapping SNPs. Only children of European ancestry were selected for further analyses. For both mothers and children, quality control was performed in PLINK (version 1.9),^29^ as previously described.^30^, ^31^ Briefly, SNPs were removed if the minor allele frequency was <1%, the Hardy–Weinberg equilibrium (HWE) *p*‐value was <1e−6 or the SNP call rate was <98%. Individual data were removed in cases of genetic and sex mismatch, excess rates of homozygosity of the genotypes (>4 SD) and genotype quality (>5% missing). After genotyping, a two‐step genotype imputation was applied for both mothers and children using the 1000 Genomes Project (phase III release version 5), build GRCh37/hg19 as reference panel, resulting in 49,008,248 SNPs. Monomorphic SNPs (with MAF < 0.5%) and SNPs with low imputation quality (*R* ^2^ < 0.3) were excluded (this includes 33,665,361 SNPs), resulting in 15,342,887 SNPs in our final imputed dataset.

### 2.6 Polygenic score (PGS) approach

For the replication part of this study, we used the publicly available GWAS summary statistics (*N* = 766,345 individuals) based on the recent study of Okbay et al.^32^ to estimate maternal PGS‐EA in our sample. This sample is overlapping with the previous study of 23andMe Research Team et al.^16^ The sample including the participants from 23andMe is not (yet) publicly available.^33^

For the extension part of this study, we used the GWAS catalog^34^ to find relevant GWAS with publicly available summary data. For the PGS‐general intelligence (PGS‐IQ) we used the study of Savage et al.,^35^ based on *N* = 269,867 individuals. For the PGS‐household income (PGS‐income) we used the study of Hill et al.,^36^ based on *N* = 505,541 individuals. See Table S1 for more details.

Maternal and child PGSs were estimated using two different methods, to investigate whether the choice of the method would influence the findings. For our main analyses, we use a PC + T (*p*‐value based clumping and thresholding) method similar to Wertz et al., using the PRSice software^37^ to estimate PGSs. For all three PGSs, the summary statistics served as the base sample, and Generation R was the target sample. The mothers and children participating in our study were never included in the base dataset. For the PGSs, only autosomal SNPs were used. PGSs were calculated using clump *r* ^2^ = 0.1, 250 kb at different *p‐*value thresholds (i.e., 0.001, 0.05, 0.1, 0.2, 0.3, 0.4, 0.5 and 1). We chose to use the best *p*‐value threshold approach for each PGS to explain the most variance using the largest *R* ^*2*^ and increase predictive power for subsequent analyses (see in Table S2 the explained variance per threshold). Since the optimal *p*‐value threshold depends on various factors, such as the effect size distribution, the power of the base and target data, the genetic architecture of the trait and the fraction of causal variants, and is thus not known a priori, this process of selecting the best *p‐*value threshold is important and comparable to tuning parameter optimization.^37^ The risk of overfitting is minimal when a large number of SNPs is used for each threshold, as has been previously discussed.^38^ However, to replicate the initial study of Wertz et al.,^39^ we have also estimated PGSs using all SNPs (*p* = 1), see Table S4.

For comparison, we used LDpred2‐auto, a version of LDpred2 that does not require a tuning sample. LDpred2 uses the same GWAS summary statistics as previously mentioned and LD information from an external LD reference sample to infer the posterior mean effect size of each SNP.^40^

### 2.7 Statistical analyses

Outliers, that is, data points deviating 3.29 SD or more from the mean, were winsorized. Maternal sensitivity and PGSs were standardized. We tested two structural models using Structural Equation Modeling (SEM) analyses to handle missing data and simultaneously estimate the effects on multiple outcomes. Models were adjusted for child sex and the first 10 principal components of genetic ancestry, to further control for hidden population stratification (see Table S3). The first model (Section 3.1.1) examined the association of maternal PGS‐EA with observed maternal sensitivity, in two developmental periods. We additionally included children's PGS‐EA to control for possible child evocative effects. The second model (Section 3.1.2) extended the replication model and examined the combined role of the strongly correlated maternal PGS‐EA, PGS‐IQ and PGS‐income. For this model as well, we added child PGSs in a second step to test for evocative gene–environment correlations. For all SEM analyses, we used the *lavaan* statistical package.^41^ We used full information maximum likelihood (FIML). The Yuan‐Bentler scaled Chi‐square estimator with Huber‐White covariance adjustment to the standard errors of each parameter estimate was used for non‐normally distributed data. Bootstrapping was used to obtain bias‐corrected confidence intervals. Model fit was assessed with the Comparative Fit Index (CFI^42^), the Tucker–Lewis Index (TLI^43^) and the Root Mean Square Error of Approximation (RMSEA^44^). Good model fit was assumed with CFI and TLI values greater than 0.95 and RMSEA smaller than 0.08.^45^ All analyses were conducted using R, version 4.04.^46^ Finally, we used linear regression to predict maternal sensitivity from maternal phenotypical education, income and IQ, and to examine the association of maternal PGSs over and above the effect of the relevant maternal phenotypes in a two‐step regression model (with maternal phenotypes added as predictors of maternal sensitivity in step 1 and maternal PGSs added in step 2). These analyses were performed in SPSS 28.0 for Windows (SPSS Inc, Chicago, IL).

## 3 RESULTS

### 3.1 Sample characteristics

Descriptive statistics and bivariate correlations between observed maternal sensitivity at two time points and maternal and child PGSs are shown in Table 1. Maternal and child PGSs were related, as expected since children receive half of their genetic variants from each parent (e.g., maternal PGS‐EA and child PGS‐EA, *r* = 0.52). The somewhat elevated genetic correlation might point to some assortative mating in our sample, which has been previously indicated for cognitive abilities.^47^, ^48^ Before conducting the main analyses, we checked whether age of the mother and sex of the child were significant covariates to include in our SEM models, but this was only the case for child sex (see Table S3).

**TABLE 1:** Descriptive statistics of and bivariate correlations between the study variables for our final sample used in the analyses.

Variable	*N*	*M*	SD	Min–Max	1	2	3	4	5	6	7	8	9
1 Age mother (at intake, in years)	1247	31.7	3.73	19.10 ‐ 43.34									
2 Maternal IQ	828	100	15.00	55.00 ‐ 120.00	**0.21**								
3 Maternal sensitivity (t)	704	0.01	0.83	−3.84 ‐ 1.88	0.03	**0.23**							
4 Maternal sensitivity (eac)	741	0.48	0.77	−1.76 ‐ 2.96	0.04	**0.28**	**0.15**						
5 Maternal PGS‐EA	1072	0.00	1.00	−3.46 ‐ 2.78	**0.27**	**0.38**	**0.11**	**0.15**					
6 Maternal PGS‐IQ	1072	0.00	1.00	−5.03 ‐ 2.29	**0.27**	**0.34**	**0.08**	**0.15**	**0.58**				
7 Maternal PGS‐Income	1072	0.00	1.00	−2.87 ‐ 3.15	**0.19**	**0.29**	**0.10**	**0.14**	**0.68**	**0.41**			
8 Child PGS‐EA	968	0.00	1.00	−3.90 ‐ 2.73	**0.24**	**0.32**	**0.10**	**0.19**	**0.52**	**0.50**	**0.37**		
9 Child PGS‐IQ	968	0.00	1.00	−3.68 ‐ 1.98	**0.22**	**0.31**	**0.08**	**0.17**	**0.44**	**0.73**	**0.39**	**0.64**	
10 Child PGS‐Income	968	0.00	1.00	−3.39 ‐ 3.21	**0.18**	**0.29**	0.02	**0.12**	**0.43**	**0.36**	**0.30**	**0.72**	**0.46**

*Note*: Significant correlations (*p* < .05) are shown in bold, t = toddlerhood, eac = early childhood, EA = educational attainment, IQ = general intelligence.

#### 3.1.1 The replication model

**FIGURE 1:**
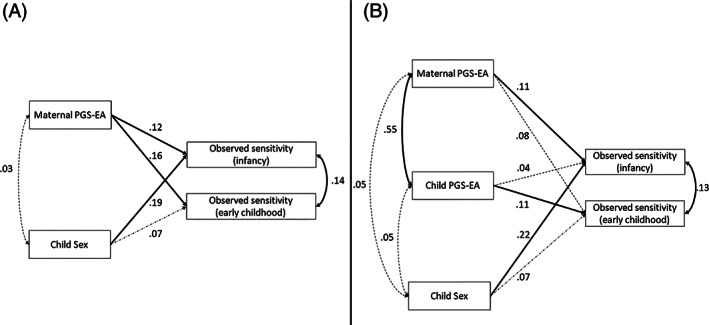
(A) Graphical representation of the replication model. This model tests the associations between the maternal polygenic score of educational attainment (PGS‐EA) and observed parenting in two developmental periods (toddlerhood and early childhood). (B) Graphical representation of the replication model controlling for child PGS‐EA. Single‐headed arrows represent regression coefficients and double‐headed arrows are correlation coefficients. Statistically significant estimates (*p* < 0.05) are shown with solid lines.

Figure 1A displays the SEM results for the replication model. Parameter estimates and bootstrapped confidence intervals of the model are presented in Table 2. In this multivariate model higher scores on maternal PGS‐EA predicted higher observed sensitivity in toddlerhood (*b* = 0.12, 95% CI 0.03, 0.20) and early childhood (*b* = 0.16, 95% CI 0.08, 0.24). The model explained 2.0% of the variance in observed sensitivity in toddlerhood and early childhood. Child sex (being a girl) was associated with more observed maternal sensitivity in toddlerhood (*b* = 0.19, 95% CI 0.04, 0.33), but not in early childhood.

**TABLE 2:** Parameter estimates of the structural equation models.

	Predictors	*b*	se	*z*	*p*	CI		*R* ^2^
*Replication model*: *Χ* ^2^ (0) = 00.00, CFI = 1.000, TLI = 1.000, RMSEA = 0.000								
Sensitivity (t)	**Maternal PGS‐EA**	0.12	0.04	2.65	0.008	0.03	0.20	0.02
	**Child sex**	0.19	0.08	2.46	0.014	0.04	0.33	
Sensitivity (eac)	**Maternal PGS‐EA**	0.16	0.04	4.04	0.000	0.08	0.24	0.02
	Child sex	0.07	0.07	1.01	0.314	−0.07	0.22	
*Replication model + child PGS‐EA*: *Χ* ^2^ (0) = 00.00, CFI = 1.00, TLI = 1.00, RMSEA = 0.000								
Sensitivity (t)	**Maternal PGS‐EA**	0.11	0.05	2.22	0.027	0.01	0.21	0.03
	Child PGS‐EA	0.04	0.05	0.92	0.357	−0.05	0.13	
	Child sex	0.22	0.08	2.82	0.005	0.07	0.38	
Sensitivity (eac)	Maternal PGS‐EA	0.08	0.05	1.53	0.126	−0.02	0.18	0.03
	**Child PGS‐EA**	0.11	0.05	2.27	0.023	0.02	0.21	
	Child sex	0.07	0.07	0.94	0.346	−0.08	0.21	
*EDINQ model*: *Χ* ^2^ (7) = 6.42, *p* = 0.49, CFI = 1.00, TLI = 1.00, RMSEA = 0.000								
EDINQ Mother	Maternal PGS‐EA	1.00				1.00	1.00	0.80
	Maternal PGS‐IQ	0.40	0.03	15.43	0.000	0.35	0.45	0.25
	Maternal PGS‐Income	0.89	0.05	18.63	0.000	0.80	0.99	0.55
Sensitivity (t)	**EDINQ Mother**	0.15	0.05	2.88	0.004	0.05	0.26	0.02
	**Child sex**	0.18	0.07	2.38	0.017	0.03	0.32	
Sensitivity (eac)	**EDINQ Mother**	0.22	0.05	4.31	0.000	0.12	0.31	0.03
	Child sex	0.08	0.07	1.06	0.290	−0.07	0.22	
*EDINQ model + child EDINQ*: *Χ* ^2^ (21) = 62.01, *p* < 0.00, CFI = 0.985, TLI = 0.974, RMSEA = 0.036								
EDINQ Mother	Maternal PGS‐EA	1.00	NA	NA	NA	1.00	1.00	0.84
	Maternal PGS‐IQ	0.39	0.02	16.30	0.000	0.34	0.43	0.25
	Maternal PGS‐Income	0.84	0.04	22.96	0.000	0.77	0.92	0.52
EDINQ Child	Child PGS‐EA	1.00	NA	NA	NA	1.00	1.00	0.79
	Child PGS‐IQ	0.69	0.04	17.42	0.000	0.61	0.77	0.34
	Child PGS‐Income	0.81	0.04	20.19	0.000	0.73	0.89	0.79
Sensitivity (t)	**EDINQ Mother**	0.14	0.07	1.96	0.049	0.00	0.28	0.02
	EDINQ Child	0.01	0.07	0.15	0.883	−0.13	0.15	
	**Child sex**	0.18	0.07	2.37	0.018	0.03	0.32	
Sensitivity (eac)	EDINQ Mother	0.10	0.07	1.32	0.187	−0.05	0.25	0.04
	**EDINQ Child**	0.16	0.08	2.03	0.043	0.01	0.31	
	Child sex	0.07	0.07	0.98	0.328	−0.07	0.21	

*Note*: Nmax = 1247, *b* = unstandardized parameter estimate, se = standard error, *z* = *Z*‐statistic, CI = confidence interval, t = toddlerhood, eac = early childhood; child sex was coded as male = 1 and female = 2; final model fits displayed in the table; *Model fit before modifications: *Χ* ^2^ (22) = 158.94, *p* < 0.01, CFI = 0.949, TLI = 0.917, RMSEA = 0.112; acceptable model fit was obtained after 1 modification: adding correlations between maternal PGS‐IQ and child PGS‐IQ (mi = 89.08, epc = 0.13); both replication models are saturated, meaning that the number of free parameters is equal to the number of variances and unique covariances, which is why fit indices are not useful for these models. Bold indicates significance values of *p* < 0.05.

We added the child's PGS‐EA to control for the genetic effects of the child. Figure 1B shows the SEM results for this model, and Table 2 summarizes the estimates of the model. Child PGS‐EA was not associated with maternal sensitivity in toddlerhood (*b* = 0.04, 95% CI −0.05, 0.13) but it was significantly associated with maternal sensitivity in early childhood (*b* = 0.11, 95% CI 0.02, 0.21). Maternal PGS‐EA was no longer significantly associated with sensitive parenting after accounting for child PGS‐EA in early childhood (*b* = 0.08, 95% CI −0.02, 0.18). *R* ^2^ increased from 2% in the maternal PGS‐EA‐only model to 3% in the model including the child PGS‐EA. Sensitivity analyses using the maternal and child PGSs with a *p*‐value of 1 showed similar results and are presented in Table S4. Sensitivity analyses using LDpred2‐auto confirmed that higher maternal PGS‐EA is associated with more maternal sensitivity in both toddlerhood and early childhood (see Table S5). However, child PGS‐EA estimated using LDpred2‐auto was not significantly associated with maternal sensitivity, in neither of the two time points.

#### 3.1.2 The EDINQ model

**FIGURE 2:**
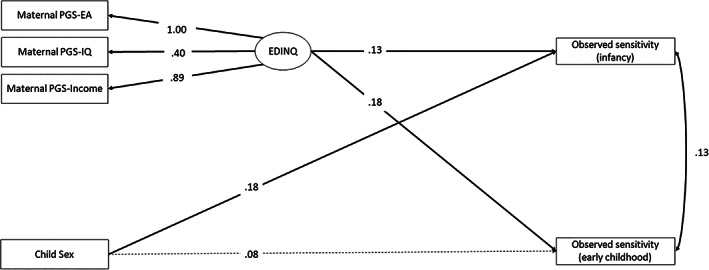
Graphical representation of the EDINQ model, combining maternal PGS‐EA, PGS‐IQ and PGS‐income in a latent factor. Observed variables are in rectangles and the latent variable is in a circle. Single‐headed arrows represent regression coefficients. Statistically significant estimates (*p* < 0.05) are shown in solid lines.

Figure 2 shows the SEM results for the EDINQ model, combining PGSs for educational attainment, income and IQ. A latent factor was estimated by combining the highly correlated maternal PGS‐EA, PGS‐IQ and PGS‐income. Table 2 summarizes the parameter estimates. In this model, the latent factor was associated with maternal sensitivity in toddlerhood (*b* = 0.15, 95% CI 0.05, 0.26). Higher scores on the latent factor were also associated with higher maternal sensitivity in early childhood (*b* = 0.22, 95% CI 0.12, 0.31). The model explained 3% of the variance in observed sensitivity in early childhood. In supplementary analyses, we conducted multiple regressions with the three PGSs as independent predictors and results were comparable (see Table S6).

**FIGURE 3:**
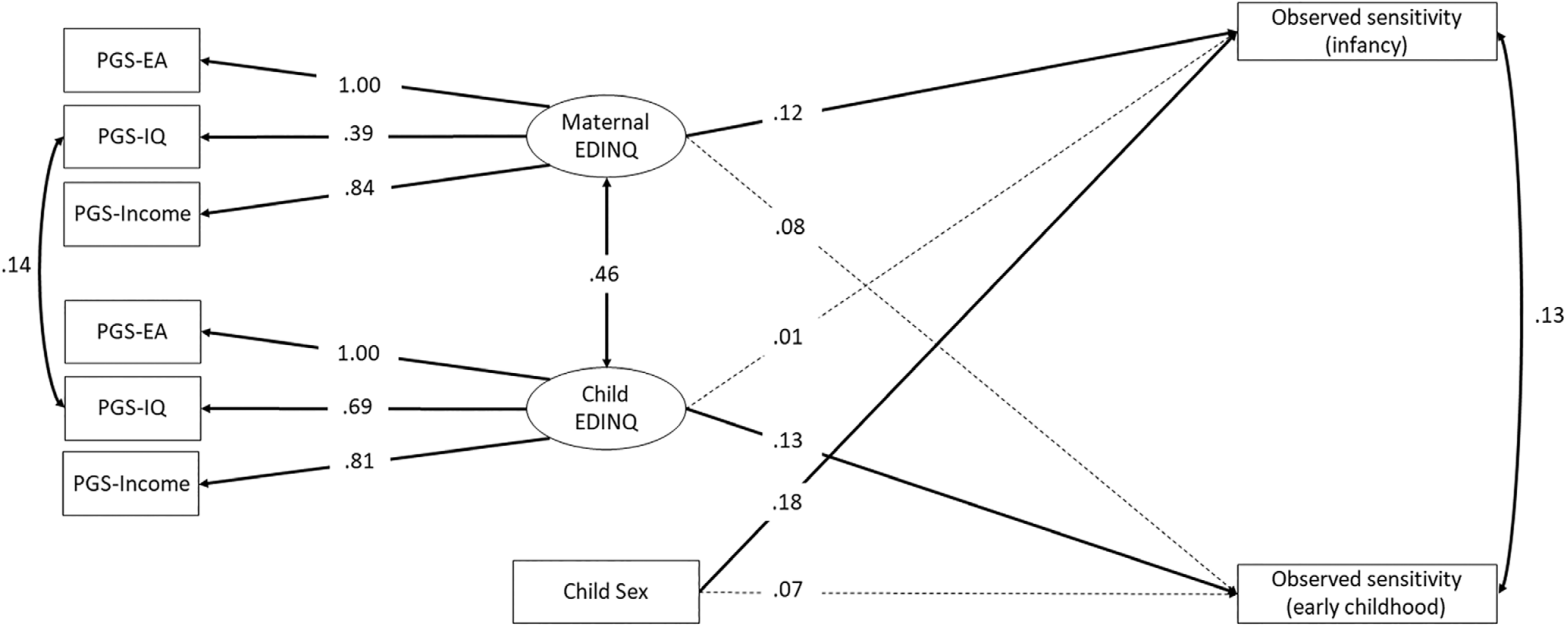
Graphical representation of the EDINQ model controlling for child PGSs, estimated in a similar way as for mothers. Observed variables are in rectangles and the latent variable is in a circle. Single‐headed arrows represent regression coefficients. Statistically significant estimates (*p* < 0.05) are shown in solid lines.

Next, a latent EDINQ factor based on children's PGS‐EA, PGS‐IQ and PGS‐income was estimated and added to the model to investigate child genetic effects. For this model, the model fit was not acceptable (*Χ* ^2^ (22) = 158.94, *p* < 0.01, CFI = 0.949, TLI = 0.917). Therefore, based on modification indices, we added the correlation between the maternal PGS‐IQ and the child PGS‐IQ (mi = 89.08, epc = 0.13) which resulted in an acceptable model fit (*Χ* ^2^ (21) = 62.01, *p* < 0.01, CFI = 0.985, TLI = 0.974, RMSEA = 0.036). The latent factor of the child PGSs was not associated with maternal sensitivity in toddlerhood (*b* = 0.01, 95% CI −0.13, 0.15). However, it was significantly associated with maternal sensitivity in early childhood (*b* = 0.16, 95% CI 0.01, 0.31) and the effect of the maternal latent factor was no longer significant (see Table 2 and Figure 3). *R* ^2^ increased from 3% in the maternal‐only EDINQ model to 4% in the model including the child PGSs in early childhood. Sensitivity analyses using the maternal and child PGSs with a *p*‐value of 1 showed similar results and are presented in Table S4. Similar to the replication model, sensitivity analyses using LDpred2‐auto confirmed that the latent factor of maternal PGS‐EDINQ was associated with more maternal sensitivity in both toddlerhood and early childhood (see Table S5). However, the child latent factor PGS‐EDINQ estimated using LDpred2‐auto was not significantly associated with maternal sensitivity, at neither of the two time points.

### 3.2 Predictive power of PGSs controlling for maternal phenotypical education, income and IQ

Table 3 presents the association between maternal phenotypes and maternal sensitivity in toddlerhood. Similar to the PGS‐models, higher maternal education was associated with more maternal sensitivity in toddlerhood (*β* = 0.11, *p* = 0.01) and early childhood (*β* = 0.19, *p* = 0.001). In early childhood, higher household income was also associated with more maternal sensitivity (*β* = 0.10, *p* = 0.02). Maternal IQ was not associated with maternal sensitivity, neither in toddlerhood nor in early childhood.

**TABLE 3:** Stepwise regression analyses testing the association between maternal phenotypes and observed maternal sensitivity in two developmental periods.

		*β*	*p*	Adjusted *R* ^2^	*F*
*In toddlerhood/replication model*					
Step 1	**Maternal education**	0.11	0.01	2.2%	*F*(3,566) = 5.24, *p* = 0.001
	Household income	0.07	0.11		
	Maternal IQ	0.05	0.24		
Step 2	Maternal PGS‐EA	0.04	0.35	2.2%	*F*(4, 565) = 4.15, *p* = 0.003
*In toddlerhood/EDINQ model*					
Step 1	**Maternal education**	0.11	0.01	2.2%	*F*(3,566) = 5.24, *p* = 0.001
	Household income	0.07	0.11		
	Maternal IQ	0.05	0.24		
Step 2	Maternal PGS‐EDINQ	0.06	0.19	2.3%	*F*(4, 565) = 4.36, *p* = 0.002
*In early childhood/replication model*					
Step 1	**Maternal education**	0.19	0.001	7.5%	*F*(3,613) = 17.74, *p* < 0.001
	**Household income**	0.10	0.02		
	Maternal IQ	0.08	0.06		
Step 2	**Maternal PGS‐EA**	0.09	0.04	8.0%	*F*(4, 612) = 14.46, *p* < 0.001
*In early childhood/EDINQ model*					
Step 1	**Maternal education**	0.19	0.001	7.5%	*F*(3,615) = 17.93, *p* < 0.001
	**Household income**	0.10	0.02		
	Maternal IQ	0.08	0.06		
Step 2	**Maternal PGS‐EDINQ**	0.10	0.01	8.4%	*F*(4, 614) = 15.19, *p* < 0.001

*Note*: Bold indicates significance values of *p* < 0.05.

Abbreviation: EA, educational attainment.

Table 3 also presents the stepwise regression analyses for maternal sensitivity in toddlerhood, with maternal phenotypes added in step 1 and maternal PGS‐EA and latent factor PGS‐EDINQ added in step 2, respectively. Addition of maternal PGS‐EA or PGS‐EDINQ did not improve the toddlerhood model. For maternal sensitivity in early childhood, however, maternal PGS‐EA and PGS‐EDINQ increased the explained variance (adjusted *R* ^*2*^ increased from 7.5% to 8.0% and 8.4%, respectively), showing predictive power of maternal PGSs over and above the predictive role of the relevant phenotypes.

## 4 DISCUSSION

Although the heritability of parenting has been examined in twin studies in the past decades, Wertz et al.^39^ were among the first to use molecular genetics to investigate genetic effects in observed parenting. In the current study, we replicated their central finding of an association between mothers' polygenic score of educational attainment (PGS‐EA) and sensitive interactions with their children. We found that already in toddlerhood a higher PGS‐EA score was associated with higher sensitivity, suggesting that genetic differences shape phenotypic differences in parenting behavior at an early stage. The association between maternal PGS‐EA and observed parenting further implies that cognitive (i.e., problem solving) and conative (i.e., planning skills, task persistence or delay of gratification and stress regulatory abilities) processes may play an important role in shaping parenting, although the exact mechanisms are as yet unknown. It is important to note however that cognitive and noncognitive skills are often interlinked, and our ability to differentiate between them is limited.

An important contribution of our study is that we showed that differences in parenting are partly explained by genetic differences between children, as previously indicated.^15^, ^49^, ^50^ The association between maternal PGS‐EA and observed sensitivity in early childhood was nullified when controlling for child PGS‐EA. The inclusion of child genetic effects in the model increased the explained variance from 2% to 3%, highlighting an important path between child genotype and parental behavior. As proposed in Belsky's^51^ process model of parenting, child influences on parent–child interactions should not be neglected. Interestingly, we did not find child genetic effects on maternal sensitivity when measured in toddlerhood. Presumably, child temperament might only begin to exert effects on maternal sensitivity after toddlerhood.^52^

Our finding has several implications. First, parental sensitivity has mainly been considered as a parental trait,^53^ predictable from pre‐birth and more or less independent of child factors.^54^ Highly sensitive parenting is thought to compensate for difficult‐to‐handle features of the child, for example, temperamental irritability or reactivity.^12^ However, moderately sensitive parents may respond more sensitively to easy‐going children than to irritable children that require more patience in searching for the right response when distressed. The child's temperament or other features might create or increase a gap between parental competence and parenting performance.

Second, the association of the child's PGS‐EA with maternal sensitivity at the expense of the mother's PGS‐EA provides some indication for evocative gene–environment correlation (rGE).^55^ Parental sensitive interaction is essentially dyadic and a two‐way traffic of information, signals and emotions, with parents in the lead but children as active participants. This study used mother–child genetic and phenotypic data. Since father genotype date were unavailable, our findings should be interpreted with caution. The observed direct genetic effects of the child may actually also include unmeasured paternal genetic effects on the parenting environment. To test whether our rGE interpretation is valid, larger studies with genetic data of family trios (child, mother, father) and observed parenting are needed.^55^, ^56^

We extended the original model of Wertz et al.^15^, ^39^ by including other relevant PGSs in the cognitive and conative domain. Given strong correlations between PGSs for EA, IQ and income we aggregated the three PGSs into a genetic indicator, PGS‐EDINQ. The advantage of such higher‐order aggregate might be better reliability and broader (ecological) validity, in particular when we compute a PGS‐EA or PGS‐EDINQ for offspring. Indeed, higher PGS‐EDINQ scores predicted more observed sensitivity in early childhood, with an effect size similar to that of PGS‐EA. The inclusion of child PGS‐EDINQ decreased the effect of maternal PGS‐EDINQ similarly as in the model with maternal and child PGS‐EA. Conceptually PGS‐EDINQ makes more sense in explaining parental sensitivity, because it provides a broader index of the context of parenting, although the aggregated factor did not predict substantially more variance. The loadings of maternal PGS‐EA and PGS‐income on the latent construct seem larger than the loading of PGS‐IQ. The substantial association of this latent construct (EDINQ) with observed parenting may imply that cognitive (i.e., IQ) and conative (i.e., planning skills, task persistence or delay of gratification and stress regulatory abilities) processes both play an important role in shaping parenting. But the purely cognitive problem‐solving abilities (PGS‐IQ) might play a somewhat smaller role than the noncognitive or conative components. The exact mechanisms remain however still uncharted, maybe also because cognitive and noncognitive skills are interlinked and difficult to clearly differentiate even at the genetic level.

Another contribution of this study is the inclusion of and control for relevant maternal phenotypes (i.e., maternal education, household income and maternal IQ) as predictors of maternal sensitivity. Higher income and IQ predicted more observed sensitive parenting, replicating earlier research.^19^ Yet, maternal PGS‐EA and PGS‐EDINQ increased explained variance, over and above the related phenotypes, emphasizing the role of PGSs as a valuable tool in family studies.

The current study has some limitations. First, it is limited in statistical power because of the relatively modest number of participants. However, the study was preregistered and replicated a previously published study. The replication part is thus transparent and reproducible without much leeway for researcher degrees of freedom. Furthermore, this study was based on the Generation R Focus cohort, which included observed measures of maternal sensitivity in only mothers of European ancestry. This homogeneity increases statistical power, but also implies limitations to the generalizability as the results cannot be generalized across ancestry groups. Broadening this line of research to other ancestries is crucial if we want them to benefit from such work. Second, genotypes were imputed to the 1000 Genomes reference panel. The Haplotype Reference Consortium (HRC) reference panel is larger and might be preferred in samples of European ancestry such as ours. However, for replication purposes we used a methodology as similar as possible to the original Dunedin study. Third, in this study we used a strictly statistical approach to combine the highly correlated PGSs of EA, IQ and household income, conceptually similarly to previous work,^57^, ^58^ and we added a sensitivity analysis with the three PGSs as separate predictors showing converging results. Other approaches, such as using genomic SEM to estimate a common factor among highly correlated traits and then creating PGS of the common factor, would focus on the joint genetic architecture of these traits, and eventually increase statistical power.^59^ In this study, we focused on PGSs of the broader cognitive and conative domain. Based on Belsky's model of parenting,^51^ PGSs of personality traits and psychopathology could also be of interest.

Although PGSs of the broader cognitive and conative domain have been found to predict parental sensitivity, we emphasize that this does not imply that parent's genetic make‐up is defining or determining their parenting skills. First, the prediction of parenting by polygenic scores is weak, especially compared with the prediction of parenting by numerous other factors, such as phenotypic self‐control skills of parents^39^ or phenotypic socioeconomic status of the family.^60^ The polygenic scores only explained up to 4% of the variance in parenting, which means that 96% of the variance are explained by other, most likely social and behavioral, factors. Polygenic scores might shape phenotypical traits and behaviors that in their turn predict parenting. Second, a significant prediction by a polygenic score of, for example income, does not mean that genes causally influence how much income a person will have or that the PGS for income will directly determine parenting behavior: genes make proteins, not behavior. We still need more complex sociopsychological process models of parenting.^51^ Third, despite the association between parents' genetic make‐up and their parenting behavior, we have shown in previous work that interventions can improve parenting behavior without altering genes, by changing the traits, circumstances or behaviors that mediate genetic influences on parenting.^61^

In sum, we replicated the Dunedin study on the relation between the polygenic score for educational attainment and observed sensitivity but also showed that the children's genotypic make‐up has to be taken into account. Our results point to the role of evocative gene–environment correlation in the dynamic interactions between parents and children. Future studies could explore potential influences of other individual differences on parenting, such as (genetic) differences in personality and susceptibility to mental health problems. For an integral model of parenting, larger and more powerful cohort studies are needed.

## CONFLICT OF INTEREST STATEMENT

The authors have no conflicts of interest to declare.

## Supporting information


**Data S1.** Supporting Information

## Data Availability

The data are not publicly available due to privacy or ethical restrictions. The study has an open policy in regard to collaboration with other research groups. Requests for collaboration should primarily be addressed to Prof. Dr. Vincent Jaddoe (v.jaddoe@erasmusmc.nl). These requests are discussed in the Generation R Study Management Team regarding their study aims, overlap with ongoing studies, logistic consequences and financial contributions.
